# Clinical and microbiological characteristics of abscesses in patients with malignancy and factors associated with 90-day mortality

**DOI:** 10.3389/fcimb.2026.1969735

**Published:** 2026-10-21

**Authors:** Huseyin Gencer, Fahriye Tugba Kos, Mustafa Guven, Muharrem Calıskan, Ugur Ozberk, Oznur Bal, Muhammed Bulent Akinci

**Affiliations:** 1 Department of Medical Oncology, Ankara Bilkent City Hospital, Ankara, Türkiye; 2 Department of Internal Medicine, Ankara Bilkent City Hospital, Ankara, Türkiye

**Keywords:** 90-day mortality, abscess, albumin, antimicrobial resistance, extended-spectrum beta-lactamase, malignancy, multidrug resistance, source control

## Abstract

**Introduction:**

Cancer-specific evidence on the outcomes and microbiology of abscesses is limited. We aimed to characterize these infections and evaluate factors associated with 90-day all-cause mortality.

**Methods:**

We conducted a single-center retrospective cohort study of adults with histologically confirmed malignancy and a first imaging-confirmed abscess; culture positivity was not required. All isolates came from percutaneous aspirates, were tested by an automated system, and were classified for extended-spectrum beta-lactamase (ESBL) production and multidrug resistance; culture reports were adjudicated for contamination by two clinicians. Survival was measured from the index date (aspiration in 177 and first documentation in seven), with first imaging and admission as alternative origins, and follow-up extended to 1 year.

**Results:**

Among 184 patients (median age, 62 years; 58.2% men; 53.8% stage IV), an organism was recorded in 124 (67.4%) and a probable pathogen in 108 (58.7%), of which 85 (78.7%) were gram-negative, mainly Escherichia coli (43) and Klebsiella pneumoniae (27). Phenotypic ESBL was recorded in 38 of 80 (47.5%) Enterobacterales isolates, 69 of 85 (81.2%) gram-negative isolates were multidrug- or extensively drug-resistant, and resistance to third-generation cephalosporins exceeded 80%, to piperacillin–tazobactam 65.7%, and to ertapenem 37.3%. A total of 42 patients died within 90 days; the 90-day survival was 76.6% (95% CI 69.7%–82.2%) from the index date and 77.7% from first imaging, both among patients who survived to the index date. In a Cox model stratified by abscess multiplicity and adjusted for age and stage, higher albumin at the index date was associated with a lower 90-day mortality (HR 0.37 per 0.5 g/dL, 95% CI 0.26–0.53; model-based risk 39.4% at 2.5 g/dL versus 6.8% at 3.5 g/dL). The association persisted after adjustment for intensive-care admission, organ support, corticosteroids, C-reactive protein, and procalcitonin but weakened after day 90 (HR 0.48 per 1 g/dL), whereas stage IV disease, unrelated to 90-day mortality, was associated with mortality thereafter (HR 2.65, 95% CI 1.45–4.84).

**Discussion:**

Ninety-day mortality was substantial, gram-negative organisms predominated, and resistance to these agents was frequent. Low albumin at the index date is a marker of early mortality that most likely reflects acute illness severity as well as nutritional and tumor-related factors, whereas cancer stage is associated with later mortality.

## 1 Introduction

Abscesses—including hepatic, intra-abdominal, retroperitoneal, pelvic, and selected extra-abdominal collections—are clinically important causes of morbidity and mortality. Pyogenic liver abscess is the best-characterized form, with a reported incidence of approximately 10–20 per 100,000 and evidence of increasing population burden (Jepsen et al., 2004; Dudina et al., 2026). Patients with malignancy are particularly vulnerable because tumor-related obstruction or necrosis, anticancer treatment, and invasive procedures can compromise host defenses and tissue integrity (Yeh et al., 1998; Chen et al., 2011). Conversely, pyogenic liver abscess itself may herald an occult malignancy, underscoring the close link between these two conditions (Lai et al., 2012).

Published cancer-associated abscess series have focused mainly on the liver. In these studies, malignancy was associated with a higher longer-term mortality, while outcomes also varied with infection source, microbiology, and the adequacy of drainage (Yeh et al., 1998; Chen et al., 2011; Lai et al., 2013; Dudina et al., 2026). Recent liver-abscess cohorts have described pathogen distribution and resistance phenotypes in general populations (Zhao et al., 2026), and patients with cancer carry a high burden of multidrug-resistant gram-negative infection (Gudiol et al., 2011), yet isolate-level resistance data for abscesses across anatomical sites in an oncology population with a defined short-term endpoint are scarce.

Admission blood tests may also reflect the host’s condition at the time of source control. The hemoglobin–albumin–lymphocyte–platelet (HALP) score has been associated with prognosis in several solid tumors (Jiang et al., 2016; Farag et al., 2023; Qian et al., 2025), but its calculation is sensitive to unit scaling and its value during an acute infection is uncertain. A component such as serum albumin may be more interpretable because it captures both nutritional status and systemic inflammation (McMillan, 2009).

We therefore aimed to describe the clinical and microbiological characteristics of imaging-confirmed abscesses across anatomical sites in patients with malignancy, including isolate-level antimicrobial resistance, and to evaluate factors associated with 90-day all-cause mortality using longer follow-up to examine whether early and later mortality have different correlates. HALP was treated as an exploratory marker without a sample-specific threshold.

## 2 Materials and methods

### 2.1 Study design, setting, and ethics

We conducted a single-center retrospective cohort study at the Department of Medical Oncology, Ankara Bilkent City Hospital, a tertiary referral hospital in Ankara, Türkiye. Eligible index abscess episodes occurred from March 8, 2019 to October 31, 2025, and outcome ascertainment extended through March 16, 2026. Reporting followed the Strengthening the Reporting of Observational Studies in Epidemiology (STROBE) recommendations (von Elm et al., 2007). The Clinical Research Ethics Committee of Ankara Bilkent City Hospital approved the study (TABED 1-25-1991; December 31, 2025). No intervention was performed for research purposes, and the analysis used retrospectively collected clinical data.

### 2.2 Source database, case identification, and cohort construction

The departmental abscess database was assembled from three sources: (i) hospital-information-system diagnosis codes and free-text diagnoses containing “abscess” or “collection” among patients followed in the Department of Medical Oncology, (ii) interventional radiology records of percutaneous aspiration or drainage of a collection in these patients, and (iii) medical oncology ward admission records. The lists were merged and de-duplicated by patient identifier, and each candidate record was reviewed manually against the imaging report and clinical notes. Capture of patients managed without aspiration depends on coding and documentation, and their comprehensive ascertainment cannot be demonstrated (see the discussion on limitations).

Eligible records were those of adults aged 18 years or older with a histopathologically confirmed malignancy and an imaging-confirmed abscess, defined as (1) a fluid collection on contrast-enhanced cross-sectional imaging and (2) a clinical diagnosis of abscess by the treating team with initiation of antimicrobial therapy, confirmed (3) by percutaneous needle aspiration in 177 patients (therapeutic in 173 and diagnostic only in four); in seven patients without aspiration, the diagnosis rested on criteria (1) and (2). Culture positivity was not required. The radiology report used the word “abscess” in 65 patients (35.5%) and described a “collection” or other lesion without that word in the remainder (**Supplementary Table S3**); formal radiological adjudication was not performed. Records were ineligible if malignancy or imaging confirmation was absent, the patient was younger than 18 years, or a linkage field, index date, death date, or last-contact date was unavailable; these fields were complete. No comparison group without abscess was available. “Imaging-confirmed abscess” denotes the clinical diagnosis recorded in routine care; “pyogenic” is used only for the published literature.

The source database contained 188 records. Linkage by patient identifier, cross-checked against name and episode dates, identified four patients who each had two records: one same-day duplicate and three later episodes (11, 29, and 90 days after the first, measured between the index dates defined below). The four excess records were excluded and the first (index) episode of each patient was retained, giving 184 patients (**Figure 1**).

**Figure 1:**
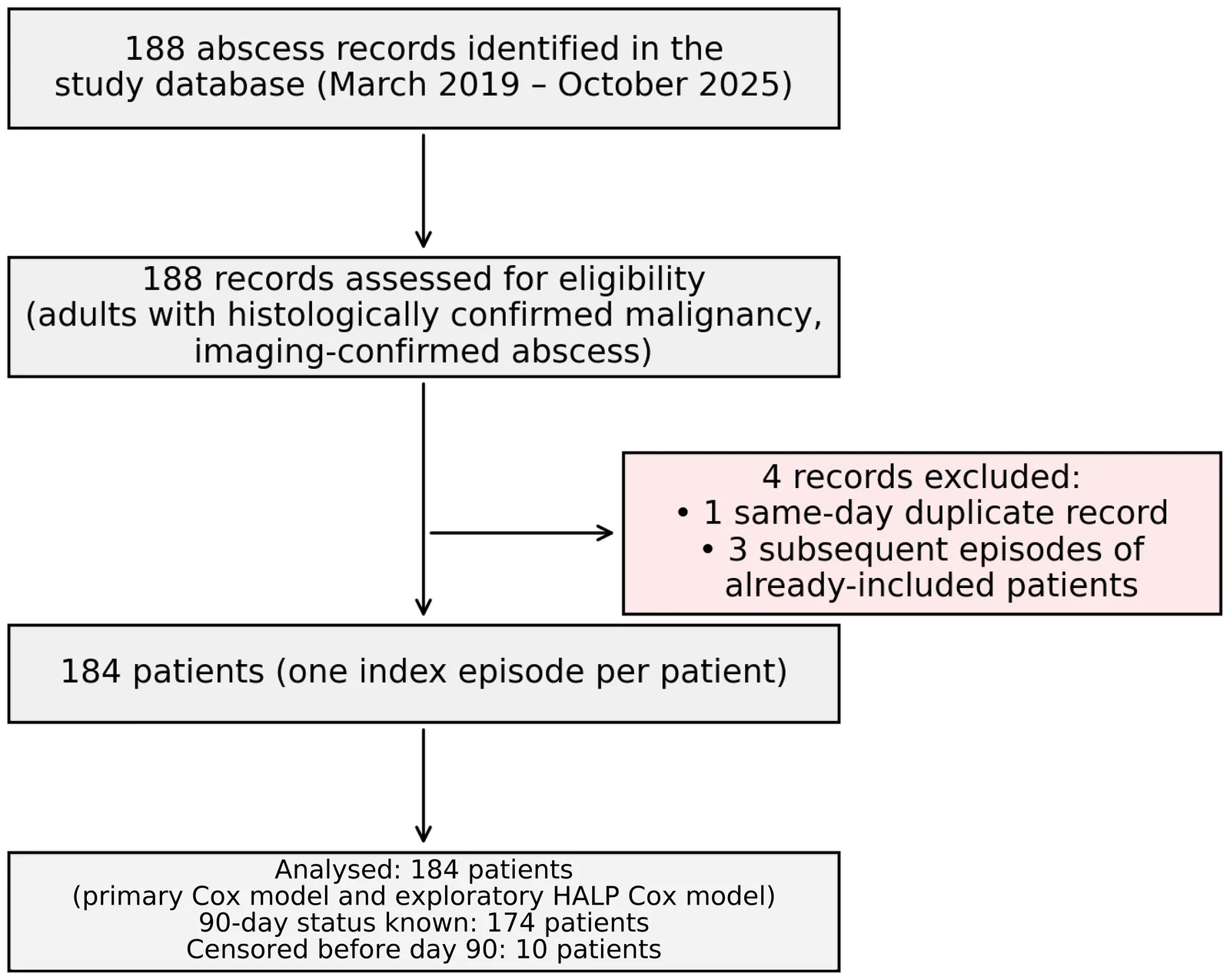
Patient flow diagram of record linkage, exclusions, and the final analytic cohort, showing 188 database records, exclusion of four records (one same-day duplicate and three subsequent episodes of already-included patients), and the final cohort of 184 patients.

The date of the index percutaneous aspiration was used as day 0 for the primary survival analyses because it is the date on which the abscess was sampled and laboratory values were obtained; for the 11 patients without therapeutic aspiration, it is the date of diagnostic aspiration (4) or first documentation (7). This date is referred to as the index date throughout; statements about measurements made at or within 2 days before the index date therefore apply to all 184 patients, including the seven who were not aspirated. The date of first diagnostic imaging and the admission date were also recovered and used as alternative time origins in sensitivity analyses.

### 2.3 Data collection, data cleaning, and clinical definitions

Demographic, oncological, and treatment variables (including the date of the last systemic therapy before the index date, corticosteroid exposure, and prior biliary or other interventional procedures), abscess characteristics, aspiration status and number of sessions, culture results, antibiotic duration, length of stay, intensive-care admission, mechanical ventilation and vasopressor use during the index hospitalization, vital status, and last-contact date were obtained from the study database. Stage IV disease was analyzed against stages I–III, recent systemic therapy was defined as treatment within 30 (and, separately, 90) days before the index date, two or more collections were classified as multiple abscesses, and two or more aspiration or drainage sessions as repeat aspiration, a measure of procedure use only. Length of stay denotes total inpatient days of the admission(s) encompassing the episode, including cancer-directed treatment.

Field-level data cleaning was performed without altering the raw source file, and data were recorded in a data-cleaning log. Five numeric values stored in date format by spreadsheet auto-conversion were verified against the source records (four abscess sizes confirmed; one antibiotic duration corrected to 15 days). Unknown free-text values were treated as missing, and two hospital stays recorded as consecutive admissions were summed. Follow-up was recalculated from the index date to death or last contact. Antibiotic duration of 0 days (28 patients) reflects incomplete documentation and was retained as recorded. All laboratory values were obtained within the 2 days preceding the index date.

All culture isolates were obtained from abscess material aspirated percutaneously at the index procedure; no blood-culture or drain-tip isolates were included. Isolates were identified and tested with an automated system interpreted according to EUCAST breakpoints in force at the time. ESBL status is the phenotypic result reported by the system, complete antibiograms were recovered for every bacterial isolate, and isolates were classified as MDR, XDR, or non-MDR according to Magiorakos et al. (2012). ESBL production is reported against the Enterobacterales denominator; one system-generated ESBL flag on an *Enterococcus faecalis* isolate was recoded as not applicable. Antibiotic-specific resistance is reported as resistant isolates over isolates tested. Resistance mechanisms were not confirmed by genotypic or confirmatory phenotypic testing; AmpC and carbapenemase production were not assessed.

Each culture report was reviewed independently by two clinicians (a medical oncologist and an internist) and adjudicated as a probable pathogen or probable contaminant on the basis of specimen type, organism identity, report wording, and the clinical record, with disagreements resolved by consensus. Three *Staphylococcus epidermidis* reports and one mixed *S. hominis*/gram-labile bacillus report were retained as probable pathogens because the organism was grown from material aspirated directly from the abscess cavity, although contamination of a skin commensal during percutaneous sampling cannot be excluded. The recorded organisms were assigned to mutually exclusive groups, and the distribution is presented with and without probable contaminants. Because the database did not distinguish no growth from a culture not obtained, episodes without a recorded organism are described as “no organism recorded”.

### 2.4 Laboratory variables and HALP calculation

Laboratory measurements obtained within the 2 days preceding the index date were used. Hemoglobin was recorded in g/dL, albumin in g/L (converted to g/dL for reporting), lymphocyte, neutrophil, and platelet counts in 10^9^/L, C-reactive protein in mg/L, and procalcitonin in µg/L. Neutropenia was defined as an absolute neutrophil count below 1.5 × 10^9^/L. To ensure consistent unit scaling, HALP was recalculated for every patient from the raw laboratory components rather than taken from the pre-existing composite column. The standard formula was as follows: HALP = hemoglobin (g/L) × albumin (g/L) × lymphocytes (10^9^/L)/platelets (10^9^/L) (Farag et al., 2023). HALP was analyzed as a continuous log_2_-transformed variable, so its effect estimate represents a twofold increase in the score; because HALP was modeled on the log scale, constant unit-scaling factors do not affect its effect estimate. No data-derived HALP threshold was calculated.

### 2.5 Outcomes and follow-up

The primary outcome was all-cause mortality within 90 days of the index abscess date. Ninety days was chosen because it is the endpoint most often used in studies of pyogenic liver abscess and of severe infection in patients with cancer, thus allowing comparison; because it captures deaths from delayed source-control failure and recurrent sepsis that occur after the first 30 days; and because all-cause mortality avoids the unreliable attribution of cause of death in patients with advanced cancer. Moreover, 30-day, 180-day, and 1-year all-cause mortality were secondary outcomes, and all available follow-up was used to examine whether the correlates of early and later mortality differ. Descriptive secondary measures included recorded-organism frequency and distribution, antimicrobial resistance, aspiration use, antibiotic duration, and length of hospital stay. Death dates and the last known contact dates were obtained from the source database. Patients who were alive at their last contact were censored on that date; follow-up for the primary survival analysis was administratively limited to 90 days.

### 2.6 Statistical analysis

Continuous variables are summarized as median with interquartile range (IQR) or range and categorical variables as number and percentage. No sample-size calculation was performed; missing values were not imputed, and denominators are stated when they differ from 184. All covariates in the primary model were complete.

Survival at 30, 90, 180, and 365 days was estimated with the Kaplan–Meier method with log–log 95% confidence intervals (CIs); median potential follow-up used the reverse Kaplan–Meier method (Schemper and Smith, 1996). Albumin below versus at least 3 g/dL was shown only in a descriptive plot; continuous albumin was used in modeling.

The primary analysis used a Cox proportional-hazards model. With 42 deaths within 90 days, complexity was restricted to three coefficients: stage IV disease, albumin, and age, prespecified because they are available for every patient and measured before the episode and not on the causal pathway between abscess and death; variables measured during the episode (C-reactive protein, procalcitonin, intensive-care admission, and organ support) may be consequences of the same process that lowers albumin and were examined only in sensitivity analyses. No univariable screening or stepwise selection was used. Abscess multiplicity showed non-proportionality on Schoenfeld residuals, and the model was stratified by single versus multiple abscesses; the retained coefficients did not violate proportionality (all *p* ≥ 0.135). Ties were handled with the Efron approximation. The model is an association model; Harrell’s C-index calculated within strata is a secondary summary, with optimism estimated from 1,000 bootstrap resamples. The albumin hazard ratio is reported per 0.5 g/dL (close to the IQR), per IQR (0.82 g/dL), and per 1 g/dL. Model-based standardized 90-day mortality at fixed albumin values was calculated by g-computation over the observed distribution of age, stage, and stratum, with a percentile bootstrap CI (500 resamples) for the risk difference between 2.5 and 3.5 g/dL. HALP (log_2_-transformed) was fitted in the identical model with albumin excluded and compared by the difference in apparent C-index, for which the bootstrap 95% CI was calculated, and by the Akaike information criterion; optimism-corrected C-indices are reported for each model separately.

Sensitivity analyses comprised alternative time origins (first diagnostic imaging, *n* = 178; admission date, *n* = 167); a 30-day endpoint; exclusion of patients without therapeutic aspiration, with *Candida* growth or with a contaminant-flagged report; a likelihood-ratio test of log-linearity (four-degrees-of-freedom B-spline); and separate addition of 17 severity, exposure, site, and microbiological variables (**Supplementary Table S1**). To examine whether the correlates of mortality change with time, the model was refitted to 180 days, 1 year, and all follow-up; a period-specific model (days 0–90 versus after day 90) was fitted by episode splitting with Wald tests for equality; and a landmark analysis of patients alive at day 90 was performed. Site-specific characteristics are reported descriptively (**Supplementary Table S4**).

The results are hazard ratios (HRs) with 95% CIs; the tests were two-sided with *p* < 0.05 considered significant, without adjustment for multiplicity because one primary model was specified. The analyses used Python 3.11 (pandas, NumPy, SciPy, and statsmodels); code and the data-cleaning log are available from the corresponding author on reasonable request.

## 3 Results

### 3.1 Patients’ characteristics

Of 188 records in the source database, one same-day duplicate and three subsequent episodes of already-included patients were excluded, leaving 184 patients (**Figure 1**). Median age was 62 years (range 33–88), 107 patients (58.2%) were men, and 106 (57.6%) had at least one recorded comorbidity. Colorectal (29.3%) and pancreatic (20.1%) cancers were the most common primary malignancies; 99 patients (53.8%) had stage IV disease. Any antitumor treatment was recorded in 139 (75.5%), systemic therapy within 30 days before the index date in 11 (6.0%), corticosteroid exposure in 41 of 177 (23.2%), a prior biliary or other interventional procedure in 44 (23.9%), and neutropenia in six (3.3%). Intensive-care admission during the index hospitalization was recorded in 61 of 178 (34.3%), vasopressor use in 30 of 176 (17.0%), and mechanical ventilation in nine (5.1%). Median albumin was 3.0 g/dL (IQR 2.7–3.5), and the standardized median HALP score was 10.5 (IQR 6.4–17.2) (**Table 1**).

**Table 1:** Demographic, oncological, treatment-exposure, and index-date laboratory characteristics (n = 184).

Characteristic	Value
Age, median (range), years	62 (33–88)
Male sex, *n* (%)	107 (58.2)
Any comorbidity, *n* (%)	106 (57.6)
Hypertension	49 (26.6)
Diabetes mellitus	45 (24.5)
Coronary artery disease	22 (12.0)
Colorectal, *n* (%)	54 (29.3)
Pancreatic, *n* (%)	37 (20.1)
Hepatobiliary, *n* (%)	23 (12.5)
Gastric, *n* (%)	16 (8.7)
Other gastrointestinal, *n* (%)	10 (5.4)
Gynecological/breast, *n* (%)	28 (15.2)
Other, *n* (%)	16 (8.7)
Stage IV disease, *n* (%)	99 (53.8)
Any antitumor treatment recorded, *n* (%)	139 (75.5)
Systemic therapy within 30 days before index date, *n* (%)	11 (6.0)
Systemic therapy within 90 days before index date, *n* (%)	34 (18.5)
Systemic corticosteroid exposure, *n* (%)	41 (23.2) [*n* = 177]
Prior biliary or other interventional procedure, *n* (%)	44 (23.9)
Neutropenia (<1.5 × 10^9^/L), *n* (%)	6 (3.3)
Intensive-care admission during index hospitalization, *n* (%)	61 (34.3) [*n* = 178]
Vasopressor use, *n* (%)	30 (17.0) [*n* = 176]
Mechanical ventilation, *n* (%)	9 (5.1) [*n* = 176]
Hemoglobin, median (IQR), g/dL	11.2 (9.3–13.1)
Albumin, median (IQR), g/dL	3.0 (2.7–3.5)
CRP, median (IQR), mg/L	90 (35–155) [*n* = 183]
Procalcitonin, median (IQR), µg/L	0.20 (0.11–0.57) [*n* = 171]
HALP, median (IQR)	10.5 (6.4–17.2)

Cancer groups are mutually exclusive and were assigned by a prespecified mapping of recorded diagnoses; one record with dual primaries (endometrial carcinoma and Klatskin tumor) was classified as hepatobiliary. Laboratory values were sampled within the 2 days preceding the index date (aspiration or, in the seven non-aspirated patients, first documentation). Percentages use the full cohort unless otherwise indicated.

IQR, interquartile range; CRP, C-reactive protein; HALP, hemoglobin–albumin–lymphocyte–platelet score.

### 3.2 Abscess characteristics and microbiology

The liver was the recorded abscess location in 73 patients (39.7%); the remaining collections were non-hepatic intra-abdominal in 66 (35.9%), retroperitoneal in 20 (10.9%), extra-abdominal (thoracic or musculoskeletal) in 11 (6.0%), pelvic in 10 (5.4%), and abdominal wall in four (2.2%). A total of 42 patients (22.8%) had multiple abscesses. The median abscess size was 7.0 cm (IQR 4.6–10.0; *n* = 176). Percutaneous needle aspiration was performed in 177 patients (96.2%): therapeutic aspiration or drainage in 173 (94.0%) and diagnostic aspiration only in four (2.2%); 52 (28.3% of the full cohort) underwent two or more therapeutic sessions. Because procedural outcomes were not recorded, these figures reflect the use of aspiration rather than its success. Median antibiotic duration was 16 days (IQR 9–26; *n* = 174), and median hospital stay was 32 days (IQR 18–47) (**Table 2**). The site-specific characteristics are given in **Supplementary Table S4**.

**Table 2:** Abscess, treatment, and microbiological characteristics (*n* = 184).

Characteristic	Value
Hepatic location, *n* (%)	73 (39.7)
Non-hepatic intra-abdominal, *n* (%)	66 (35.9)
Retroperitoneal, *n* (%)	20 (10.9)
Extra-abdominal (thoracic/musculoskeletal), *n* (%)	11 (6.0)
Pelvic, *n* (%)	10 (5.4)
Abdominal wall, *n* (%)	4 (2.2)
Multiple abscesses (≥2), *n* (%)	42 (22.8)
Abscess size, median (IQR), cm	7.0 (4.6–10.0) [*n* = 176]
Any percutaneous needle aspiration, *n* (%)	177 (96.2)
Therapeutic aspiration or drainage	173 (94.0)
Diagnostic aspiration only	4 (2.2)
≥2 aspiration/drainage sessions, *n* (%)	52 (28.3)
Organism recorded, *n* (%)	124 (67.4)
Monomicrobial gram-negative, *n* (%)^a^	85 (68.5)
Monomicrobial gram-positive, *n* (%)^a^	30 (24.2)
Monomicrobial fungal (*Candida albicans*), *n* (%)^a^	6 (4.8)
Polymicrobial, *n* (%)^a^	1 (0.8)
Unidentified, *n* (%)^a^	2 (1.6)
Adjudicated probable contaminant, *n* (%)^a^	16 (12.9)
Probable pathogen recorded, *n* (% of cohort)	108 (58.7)
Monomicrobial gram-negative, n (%)^b^	85 (78.7)
Monomicrobial gram-positive, *n* (%)^b^	16 (14.8)
Monomicrobial fungal, *n* (%)^b^	6 (5.6)
Polymicrobial, *n* (%)^b^	1 (0.9)
*E. coli*, *n*	43
*Klebsiella* spp., *n* (*K. pneumoniae*/*K. aerogenes*)	29 (27/2)
Other Enterobacterales, *n*	8
*Pseudomonas aeruginosa*/*Stenotrophomonas maltophilia*, *n*	4/1
*Enterococcus* spp., *n* (probable pathogens)	12 (10)
*Staphylococcus aureus*, *n*	3
ESBL-producing, *n*/*N* (%) of Enterobacterales	38/80 (47.5)
*E. coli*	28/43 (65.1)
*K. pneumoniae*	8/27 (29.6)
*K. aerogenes*	0/2 (0.0)
Other Enterobacterales	2/8 (25.0)
MDR, *n*/*N* (%) of gram-negative isolates	55/85 (64.7)
XDR, *n*/*N* (%) of gram-negative isolates	14/85 (16.5)
Antibiotic duration, median (IQR), days	16 (9–26) [*n* = 174]
Hospital stay, median (IQR), days	32 (18–47)

Anatomical categories are mutually exclusive; non-hepatic intra-abdominal comprises quadrant and midline collections. All isolates were obtained by percutaneous needle aspiration of the abscess. Organism-specific counts include the single polymicrobial report. ESBL testing was performed on all 80 Enterobacterales isolates (*E. coli*—43, *K. pneumoniae*—27, *K. aerogenes*—2, and eight other Enterobacterales comprising *Enterobacter cloacae* complex, *Citrobacter freundii*, *Proteus mirabilis*, *Proteus vulgaris*, and *Serratia marcescens*); the phenotype is not applicable to *Pseudomonas*, *Stenotrophomonas*, and gram-positive or fungal isolates. The denominator for aspiration-session counts is the full cohort. “No organism recorded” (*n* = 60) includes cultures with no growth and cultures not obtained, which the database did not distinguish.

IQR, interquartile range; ESBL, extended-spectrum beta-lactamase; MDR, multidrug-resistant; XDR, extensively drug-resistant (Magiorakos et al., 2012).

^a^ Percentage among the 124 patients with a recorded organism.

^b^ Percentage among the 108 patients with a probable pathogen after exclusion of 16 adjudicated contaminants.

An organism was recorded in 124 patients (67.4%), all from percutaneous aspirates; for the remaining 60, the database did not distinguish no growth from culture not obtained. Among recorded organisms, 85 (68.5%) were monomicrobial gram-negative, 30 (24.2%) monomicrobial gram-positive, six (4.8%) *Candida albicans*, one polymicrobial, and two unidentified. A total of 16 reports (12.9%) were adjudicated as probable contaminants (*Staphylococcus epidermidis*—four of seven, *S. hominis*—two, *Micrococcus* spp.—two, *Corynebacterium striatum*, *Cutibacterium acnes*, *Dermabacter hominis*, *Bacillus cereus*, one *Enterococcus faecalis*, one *E. faecium*, and two unidentified isolates). After their exclusion, 108 patients (58.7%) had a probable pathogen: 85 (78.7%) monomicrobial gram-negative, 16 (14.8%) monomicrobial gram-positive, six (5.6%) fungal, and one polymicrobial. *Escherichia coli* was identified in 43 patients, *Klebsiella* species in 29 (*K. pneumoniae* 27), other Enterobacterales in eight, *Pseudomonas aeruginosa* in four, *Enterococcus* species in 12 (10 probable pathogens), and *Staphylococcus aureus* in three (**Table 2**). *E. coli* and *K. pneumoniae* accounted for 35 of 47 (74.5%) probable pathogens in hepatic and 27 of 41 (65.9%) in non-hepatic intra-abdominal abscesses, whereas enterococci were proportionally more frequent in retroperitoneal (three of 10) and pelvic (two of six) collections (**Figure 2**).

**Figure 2:**
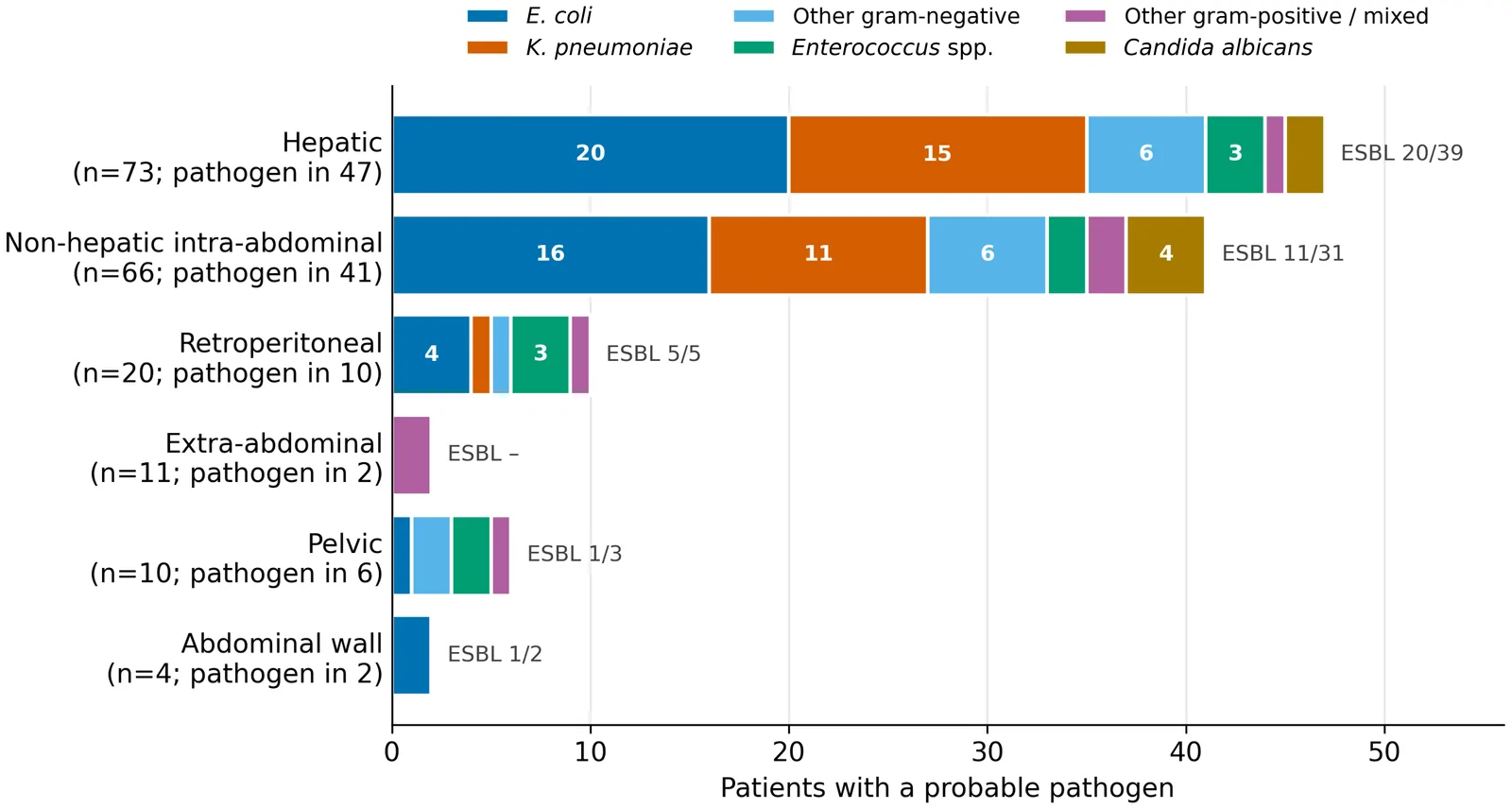
Distribution of adjudicated probable pathogens by anatomical site among 108 patients, with the number of ESBL-producing Enterobacterales isolates over the number of Enterobacterales isolates at each site. Stacked horizontal bar chart of probable pathogens by anatomical site: hepatic (47 patients; *E. coli*—20, *K. pneumoniae*—15), non-hepatic intra-abdominal (41; *E. coli*—16, *K. pneumoniae*—11), retroperitoneal (10), pelvic (six), extra-abdominal (two), and abdominal wall (two), with ESBL counts 20/39, 11/31, 5/5, 1/3, 0/0, and 1/2.

Phenotypic ESBL testing was performed on all 80 Enterobacterales isolates, and 38 (47.5%) were ESBL producers: 28 of 43 (65.1%) *E. coli*, eight of 27 (29.6%) *K. pneumoniae*, zero of two *K. aerogenes*, and two of eight (25.0%) other Enterobacterales (44.7% of all gram-negative isolates; 20.7% of the cohort). Among gram-negative isolates, 55 (64.7%) were MDR and 14 (16.5%) XDR. Resistance among Enterobacterales (**Table 3**) exceeded 80% for ceftriaxone, ceftazidime, cefepime, and amoxicillin–clavulanate, was 65.7% for piperacillin–tazobactam, 62.3% for ciprofloxacin, and 37.3% for ertapenem (any tested carbapenem 25 of 71, 35.2%; carbapenemase production was not tested), whereas amikacin (15.4%), colistin (13.6%), and tigecycline (0%) retained activity. ESBL-producing Enterobacterales were recorded in 20 of 39 hepatic, 11 of 31 non-hepatic intra-abdominal, and five of five retroperitoneal isolates (**Figure 2**; **Supplementary Table S4**).

**Table 3:** Antibiotic-specific resistance among 80 Enterobacterales isolates.

Antibiotic	Resistant/tested	% resistant
Ceftriaxone	62/73	84.9
Ceftazidime	63/76	82.9
Cefepime	54/65	83.1
Amoxicillin–clavulanate	62/75	82.7
Piperacillin–tazobactam	46/70	65.7
Ciprofloxacin	48/77	62.3
Trimethoprim–sulfamethoxazole	41/76	53.9
Gentamicin	12/32	37.5
Ertapenem	25/67	37.3
Meropenem	5/19	26.3
Imipenem	7/35	20.0
Any tested carbapenem	25/71	35.2
Amikacin	12/78	15.4
Colistin	3/22	13.6
Tigecycline	0/31	0.0

Susceptibility was determined with an automated system and interpreted according to EUCAST breakpoints; isolates reported as intermediate (susceptible, increased exposure) were not counted as resistant. Denominators differ because not every agent was tested on every isolate.

### 3.3 Ninety-day outcomes

During all available follow-up, 101 deaths were recorded; median potential follow-up by reverse Kaplan–Meier estimation was 901 days. A total of 25 deaths occurred within 30 days and 42 within 90 days. The estimated survival was 86.3% (95% CI 80.4–90.5%) at 30 days and 76.6% (95% CI 69.7–82.2%) at 90 days (**Figure 3A**). There were 10 patients censored alive before day 90. With first diagnostic imaging as the time origin (178 patients; aspiration a median of 6 days [IQR 2–12] after imaging), survival was 91.5% at 30 days and 77.7% (95% CI 70.7–83.1%) at 90 days; with the admission date (167 patients; median 9 days before the index date), it was 93.4% and 75.8%, respectively. These re-anchored analyses re-time the same patients, all of whom had by construction survived to the index date, so the interval from imaging or admission to the index date is immortal person-time; the similar 90-day estimates, therefore, indicate only that the choice of origin among the included patients had little numerical effect and do not quantify the bias arising from deaths before aspiration, which the higher 30-day estimates with the earlier origins illustrate.

**Figure 3:**
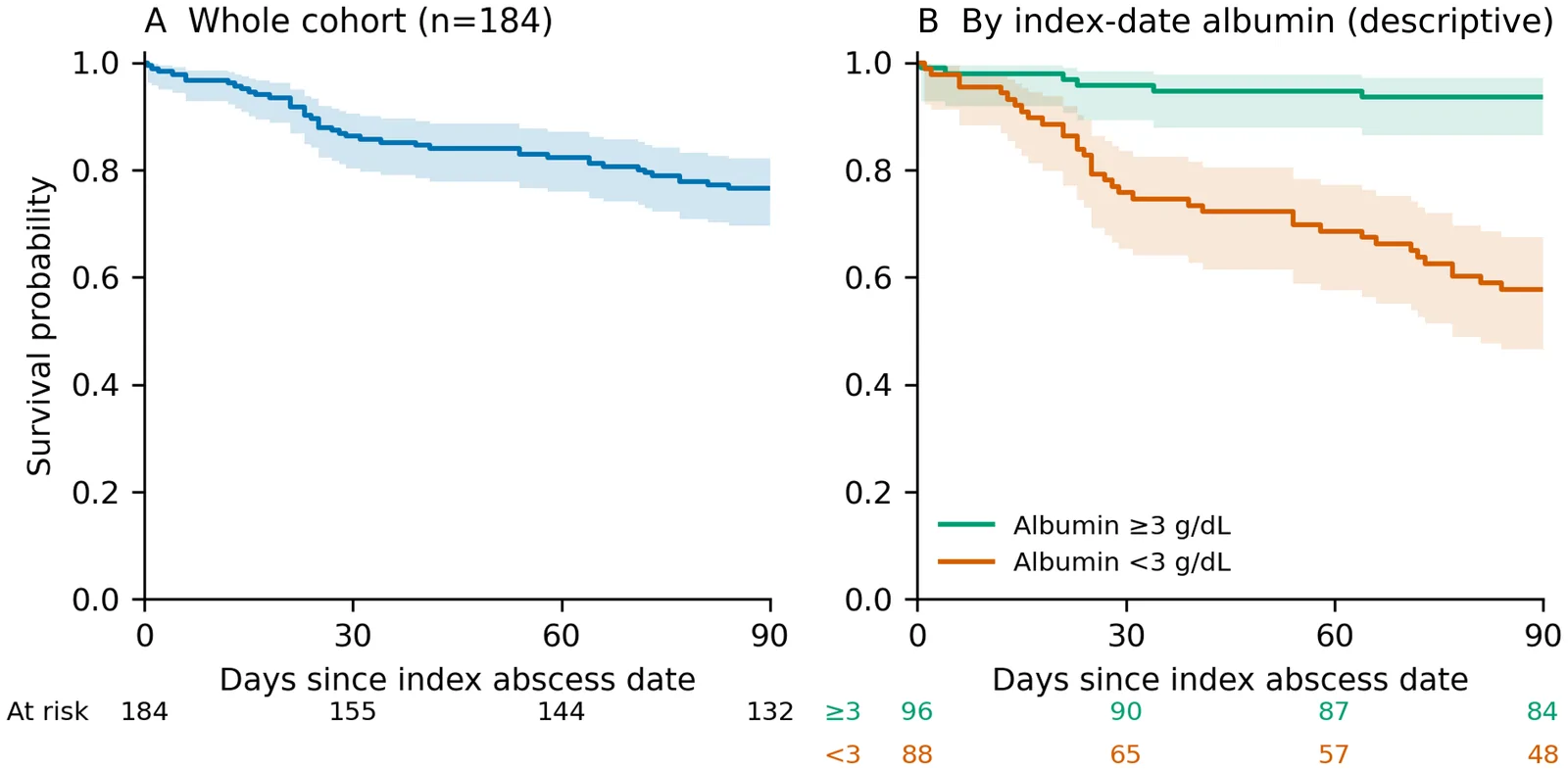
Kaplan–Meier estimates of 90-day survival with numbers at risk at 0, 30, 60, and 90 days. **(A)** Whole cohort with 95% confidence interval. **(B)** Descriptive groups defined by albumin <3 versus ≥3 g/dL. Two Kaplan–Meier survival curves over 90 days with number-at-risk tables: **(A)** shows the whole cohort of 184 patients declining to 76.6% survival; **(B)** compares albumin groups, with the low-albumin group declining to 57.8% and the high-albumin group to 93.7%.

For a descriptive presentation, 88 patients had albumin below 3 g/dL and 96 had albumin of at least 3 g/dL. The estimated 90-day survival was 57.8% (95% CI 46.5–67.4%) in the lower-albumin group and 93.7% (95% CI 86.4–97.1%) in the higher-albumin group (log-rank *p* < 0.001; **Figure 3B**). This grouping was not used to estimate the continuous albumin effect in the multivariable model. The crude Kaplan–Meier 90-day mortality was 64.0% (*n* = 25) below 2.5 g/dL, 33.2% (*n* = 63) at 2.5–2.9 g/dL, 9.3% (*n* = 44) at 3.0–3.4 g/dL, and 3.8% (*n* = 52) at 3.5 g/dL or above.

### 3.4 Multivariable analyses

In the Cox model stratified by abscess multiplicity, higher albumin was associated with lower 90-day mortality after adjustment for age and cancer stage: HR 0.37 per 0.5 g/dL (95% CI 0.26–0.53), HR 0.20 per interquartile range of 0.82 g/dL (95% CI 0.11–0.35), and HR 0.14 per 1 g/dL (95% CI 0.07–0.28; *p* < 0.001). Stage IV disease (HR 0.70, 95% CI 0.37–1.33) and age (HR 1.16 per 10 years, 95% CI 0.89–1.52) were not associated with the endpoint (**Table 4**) The apparent C-index was 0.773, and the optimism-corrected C-index was 0.762. On the absolute scale, model-based standardized 90-day mortality was 39.4% at an albumin level of 2.5 g/dL, 17.2% at 3.0 g/dL, and 6.8% at 3.5 g/dL, an absolute difference of 32.5 percentage points (bootstrap 95% CI 22.7–40.4) between 2.5 and 3.5 g/dL (**Table 4**).

**Table 4:** Primary Cox model of 90-day all-cause mortality and model-based absolute risks.

Variable	HR	95% CI	*p*
Albumin, per 0.5 g/dL	0.37	0.26–0.53	<0.001
Albumin, per interquartile range (0.82 g/dL)	0.20	0.11–0.35	<0.001
Albumin, per 1 g/dL	0.14	0.07–0.28	<0.001
Stage IV disease	0.70	0.37–1.33	0.278
Age, per 10 years	1.16	0.89–1.52	0.280
Model-based standardized 90-day mortality			
Albumin 2.0 g/dL	72.9%		
Albumin 2.5 g/dL	39.4%		
Albumin 3.0 g/dL	17.2%		
Albumin 3.5 g/dL	6.8%		
Albumin 4.0 g/dL	2.6%		
Risk difference, 2.5 versus 3.5 g/dL	32.5 percentage points	22.7–40.4	

*n* = 184, 42 events; Cox model stratified by abscess multiplicity, Efron approximation for ties. The three albumin rows are rescalings of a single coefficient. Apparent C-index 0.773; optimism-corrected 0.762 (1,000 bootstrap resamples). Standardized risks were obtained by g-computation over the observed distribution of age, stage, and stratum; the CI for the risk difference is a percentile bootstrap interval (500 resamples).

CI, confidence interval; HR, hazard ratio.

In sensitivity analyses, the albumin association was essentially unchanged with first imaging (HR 0.16 per 1 g/dL, 95% CI 0.08–0.32; 39 events) or admission (HR 0.17, 0.08–0.36) as the time origin; for the 30-day endpoint (HR 0.13, 0.05–0.32; 25 events); after exclusion of patients without therapeutic aspiration, with *Candida* growth or with a contaminant-flagged report; and after separate addition of each of 17 severity, exposure, site, and microbiological variables (HR 0.12–0.17 per 1 g/dL; **Supplementary Table S1**). None of the added variables—including intensive-care admission, vasopressor use, mechanical ventilation, C-reactive protein, procalcitonin, ESBL status, and anatomical site—was associated with 90-day mortality except neutropenia, an estimate based on six patients reported only in **Supplementary Table S1**. Median albumin was lower in patients admitted to intensive care (2.8 versus 3.2 g/dL) and in those requiring vasopressors or ventilation (2.75 versus 3.1 g/dL). There was no evidence of departure from log-linearity (*p* = 0.99). Hepatic versus non-hepatic location was not associated with 90-day mortality (univariable HR 0.85, 95% CI 0.45–1.59), although wide confidence intervals leave real differences between sites possible.

In the exploratory HALP analysis fitted in the same stratified Cox model on all 184 patients, HALP was not associated with 90-day mortality (HR 0.79 per doubling, 95% CI 0.60–1.05; *p* = 0.101); the apparent C-index of the HALP model was 0.604 versus 0.773 for the albumin model (difference in apparent C-index 0.169, bootstrap 95% CI 0.078–0.247), and the optimism-corrected C-indices were 0.572 and 0.762, respectively (**Supplementary Table S2**).

### 3.5 Longer follow-up

The estimated survival was 68.4% (95% CI 60.9–74.7%) at 180 days and 54.3% (46.4–61.5%) at 1 year (56 and 78 deaths). With follow-up extended to 180 days, 1 year, and all follow-up (101 deaths), the albumin HR per 1 g/dL attenuated from 0.14 to 0.20, 0.25, and 0.31, respectively, whereas the stage IV HR rose from 0.70 to 0.94, 1.12, and 1.43, respectively (**Table 5**). In the period-specific model, the albumin HR was 0.14 (0.07–0.28) during days 0–90 and 0.48 (0.30–0.76) thereafter (*p* = 0.004 for difference), and the stage IV HR was 0.70 (0.37–1.33) and 2.65 (1.45–4.84), respectively (*p* = 0.003). Among the 132 patients alive at day 90, stage IV disease was associated with subsequent mortality (HR 2.29, 95% CI 1.06–4.94 to 1 year), and the albumin association was attenuated (HR 0.44, 0.24–0.82). Kaplan–Meier survival at 1 year was 48.9% in stage IV versus 60.9% in stage I–III patients despite the reversed ordering at 90 days (79.5% versus 73.2%).

**Table 5:** Cox models of all-cause mortality over increasing follow-up and period-specific estimates.

Analysis	Events	Albumin, HR per 1 g/dL (95% CI)	Stage IV, HR (95% CI)	Age, HR per 10 years (95% CI)
90 days (primary)	42	0.14 (0.07–0.28)	0.70 (0.37–1.33)	1.16 (0.89–1.52)
180 days	56	0.20 (0.11–0.35)	0.94 (0.54–1.65)	1.12 (0.89–1.40)
1 year	78	0.25 (0.16–0.39)	1.12 (0.70–1.80)	1.05 (0.87–1.27)
All follow-up	101	0.31 (0.21–0.45)	1.43 (0.94–2.18)	1.07 (0.90–1.27)
Period-specific, days 0–90	42	0.14 (0.07–0.28)	0.70 (0.37–1.33)	1.16 (0.89–1.52)
Period-specific, after day 90	59	0.48 (0.30–0.76)	2.65 (1.45–4.84)	0.95 (0.76–1.18)
Landmark (alive at day 90; *n* = 132) to 1 year	36	0.44 (0.24–0.82)	2.29 (1.06–4.94)	0.90 (0.68–1.18)

All models are stratified by abscess multiplicity (*n* = 184 unless stated). The period-specific model was fitted by episode splitting at day 90; Wald tests for equality of the early and late coefficients: albumin *p* = 0.004, stage IV *p* = 0.003. Optimism-corrected C-indices: 0.762 (90 days), 0.736 (180 days), 0.711 (1 year), and 0.700 (all follow-up).

CI, confidence interval; HR, hazard ratio.

## 4 Discussion

In this single-center cohort of 184 patients with malignancy and an imaging-confirmed abscess, sampled by percutaneous aspiration in 177 (96.2%), 42 deaths occurred within 90 days and the estimated 90-day survival was 76.6%. Enteric gram-negative organisms dominated the recorded microbiology; nearly half of Enterobacterales isolates produced ESBL, four-fifths of gram-negative isolates were MDR or XDR, and resistance to third-generation cephalosporins, piperacillin–tazobactam, and ertapenem was frequent. A lower albumin level at the index date was associated with 90-day mortality after adjustment for age and cancer stage, and the association held across sensitivity analyses that included intensive-care admission and organ support; with longer follow-up, it attenuated, while cancer stage—unrelated to mortality in the first 90 days—became the dominant correlate of later death.

Most previous cohorts evaluated pyogenic liver abscess alone (Yeh et al., 1998; Chen et al., 2011; Lai et al., 2013; Mukthinuthalapati et al., 2020; Rossi et al., 2022; Dudina et al., 2026), and a recent etiological analysis described pathogen distribution and susceptibility in a general population (Zhao et al., 2026). The present study followed an oncology population across hepatic and non-hepatic sites, restricted the analysis to each patient’s first episode, analyzed isolate-level microbiology from abscess aspirates with contamination adjudication, and specified the 90-day endpoint in advance.

The short-term endpoint changes the interpretation of cancer stage. Prior studies reported worse longer-term outcomes in patients with malignancy or advanced disease (Yeh et al., 1998; Chen et al., 2011; Dudina et al., 2026), whereas stage IV disease was not associated with 90-day mortality here, and the point estimate was below unity. First, acute physiological reserve and infection severity may dominate very early risk while cancer stage becomes influential later; the extended analyses support this directly since the stage IV hazard ratio rose from 0.70 in the first 90 days to 2.65 thereafter, and 1-year survival was clearly lower in stage IV patients. Second, conditioning cohort entry on the occurrence of an abscess can induce index-event (collider) bias: factors that increase the probability of an abscess in less advanced disease—biliary intervention, curative-intent surgery, and intensive systemic treatment—are over-represented among stage I–III patients and may carry their own early mortality, so the early stage IV estimate cannot be read as protective. Nearly a quarter of the cohort had undergone a prior biliary or interventional procedure, consistent with this mechanism.

Albumin is a negative acute-phase reactant as well as a marker of nutritional and disease burden. Because all samples were obtained within 2 days of the index date—for aspirated patients, a median of 6 days after first imaging and 9 days after admission—albumin here was measured during the established infectious episode and should be read as a marker of the combined nutritional, tumor-related, and acute inflammatory state at the time of source control rather than of baseline host reserve; its association with mortality cannot be assigned to malnutrition alone and is not necessarily causal (McMillan, 2009). The large magnitude of the association itself suggests that albumin captures acute illness severity: patients admitted to intensive care or requiring organ support had lower albumin, and although adjustment for these variables, C-reactive protein, and procalcitonin did not attenuate the estimate—they are coarse, so residual confounding by severity is likely; data on performance status, sepsis scores, antimicrobial timing, and source-control delay were unavailable. Albumin is routinely available and may help identify patients who require closer assessment; it is an association, not a validated prediction rule. HALP, fitted in the same model on the same patients, showed no association with 90-day mortality and lower discrimination than albumin, in contrast to tumor-prognosis studies (Jiang et al., 2016; Farag et al., 2023; Qian et al., 2025) whose populations and outcomes are not directly comparable.

The recorded microbiology was dominated by monomicrobial gram-negative growth, with *E. coli* and *K. pneumoniae* accounting for three quarters of probable pathogens in hepatic abscesses, consistent with reports in pyogenic liver abscess (Chen et al., 2011; Siu et al., 2012; Zhao et al., 2026), whereas enterococci were proportionally more frequent in retroperitoneal and pelvic collections. The resistance findings are the most clinically salient descriptive result: ESBL production in 47.5% of Enterobacterales (65.1% of *E. coli*), MDR or XDR phenotypes in 81.2% of gram-negative isolates, and resistance to piperacillin–tazobactam in two-thirds and to ertapenem in more than a third of Enterobacterales. These proportions are in keeping with the burden of resistant gram-negative infection in patients with cancer (Gudiol et al., 2011) who are exposed to repeated hospitalization, antimicrobial treatment, and biliary or gastrointestinal instrumentation. Empirical regimens based on third-generation cephalosporins or piperacillin–tazobactam would have failed to cover most Enterobacterales isolates and ertapenem more than one-third, whereas amikacin retained activity against 85%. The pattern of phenotypes—resistance to third-generation cephalosporins in more than 80% of Enterobacterales, while a phenotypic ESBL result was recorded in 47.5%, together with resistance to at least one carbapenem in 35.2%—implies that mechanisms other than ESBL, notably AmpC β-lactamases and, in particular, carbapenemases, contributed to the observed resistance; because the automated-system ESBL phenotype is unreliable in carbapenemase-producing isolates, the ESBL proportion should be read as approximate, and resistance mechanisms were not genotypically confirmed. Because the cohort is a single-center series with probable ascertainment through interventional referral, these figures describe local epidemiology in a selected population and cannot by themselves support specific empirical regimens, but they indicate that local resistance surveillance should inform empirical therapy in this population. Neither ESBL nor MDR/XDR status was associated with 90-day mortality, although the number of events limits this comparison.

Therapeutic percutaneous aspiration or drainage was recorded in 94.0% of patients and any aspiration in 96.2%, consistent with its central role in management (Yu et al., 2004; Lai et al., 2013; Mukthinuthalapati et al., 2020; Justo et al., 2023). Aspiration frequency cannot be equated with effective source control, and the very high rate probably partly reflects case ascertainment since capture of conservatively or surgically managed patients depended on coding and documentation. Without radiological resolution or re-intervention data, drainage strategies cannot be compared, and the long median hospital stay reflects a tertiary oncology setting in which admissions encompass cancer-directed treatment.

Several limitations should be emphasized. The retrospective single-center design is vulnerable to selection, measurement, and referral bias; ascertainment of patients managed without aspiration cannot be demonstrated, and the findings apply mainly to patients whose abscess was confirmed and sampled by aspiration (177 of 184). The cohort combined anatomically heterogeneous abscesses and had no cancer control group without abscess. The case definition was not radiologically adjudicated; the report used the word “abscess” in only about one-third of patients, and sterile collections, bilomas, or necrotic trumor cannot be excluded, particularly in non-aspirated patients and in those without a recorded organism. Six *Candida* episodes were retained, and 10 patients were censored before 90 days. The index date is the date of aspiration in 177 patients (a median of 6 days after first imaging) and of first documentation in seven; patients dying before the index date could not enter the cohort, so early survival is likely to be somewhat overestimated; the analyses with earlier time origins include only patients who survived to the index date and, therefore, cannot quantify this bias. Performance status, sepsis scores, antimicrobial timing and appropriateness, source-control delay, and detailed trumor burden were unavailable; intensive-care admission and organ support are coarse; antibiotic duration was incompletely documented in 28 patients. Contamination adjudication was clinical; resistance mechanisms were not genotypically confirmed, and the automated ESBL phenotype may misclassify AmpC- or carbapenemase-producing isolates; and episodes without a recorded organism could not be separated into culture-negative versus culture-not-obtained. Cause-specific death, radiological resolution, and recurrence were not analyzed. The models were internally bootstrapped but not externally validated.

Prospective multicenter studies with explicit case ascertainment, radiological adjudication, confirmatory resistance testing, sepsis-severity and source-control variables, cause-specific mortality, and external validation are needed; until then, the findings are best regarded as descriptive and hypothesis-generating.

## 5 Conclusions

Among adults with malignancy and imaging-confirmed abscesses, most of which were sampled by percutaneous aspiration, 90-day mortality was substantial. Enteric gram-negative organisms predominated; ESBL production was recorded in nearly half of Enterobacterales isolates, most gram-negative isolates were MDR or XDR, and resistance to third-generation cephalosporins, piperacillin–tazobactam, and ertapenem was frequent, so local resistance data deserve attention when treatment is planned, although no preferred empirical regimen can be derived from this study. Lower albumin at the index date was associated with early mortality after adjustment for age and cancer stage; because it most likely reflects acute illness severity as well as nutritional and tumor-related factors, it should be read as a readily available marker of short-term risk awaiting external validation rather than an established prognostic factor. Cancer stage, unrelated to mortality within 90 days, was the dominant correlate of mortality thereafter.

## Data Availability

The datasets presented in this article are not readily available because they contain potentially re-identifiable clinical data. A de-identified minimal dataset, the data-cleaning log, and the analysis code may be made available by the corresponding author upon reasonable request and subject to institutional approval. Requests to access the datasets should be directed to Huseyin Gencer, huseyinrahmigencer@gmail.com.

## Supplementary material

Supplementary Material 1 Completed STROBE checklist for cohort studies.

Supplementary Material 2 **Supplementary Table S1**—Full results of the sensitivity analyses of 90-day all-cause mortality, including alternative time origins; **Supplementary Table S2**—HALP fitted in the primary Cox model; **Supplementary Table S3**—Wording of the index radiology report; **Supplementary Table S4**—Site-specific characteristics, microbiology and outcomes.
