# Supplementary material for: Clinical and microbiological characteristics of abscesses in patients with malignancy and factors associated with 90-day mortality
Source: Front Cell Infect Microbiol. 2026 Oct 21;16:1969735. doi: 10.3389/fcimb.2026.1969735 (PMC13730854; doi:10.3389/fcimb.2026.1969735)
Supplement: Supplementary file 1 [file SupplementaryFile1.pdf]

Supplementary Material 2

Table S1. Sensitivity analyses of the primary Cox model of 90-day all-cause mortality

All models are stratified by abscess multiplicity and adjusted for stage IV disease and age (per 10 years) unless stated. Albumin HR is per 1 g/dL.

Analysis	n	Events	Albumin HR (95% CI)	Added variable HR (95% CI), p
Primary model (index date as day 0; aspiration in 177 patients, first documentation in 7)	184	42	0.14 (0.07–0.28)	—
**Alternative time origins**				
First diagnostic imaging date as day 0	178	39	0.16 (0.08–0.32)	stage IV 0.70 (0.36–1.35); age 1.13 (0.85–1.49)
Admission date as day 0	167	39	0.17 (0.08–0.36)	stage IV 0.66 (0.34–1.28); age 1.12 (0.85–1.47)
**Alternative endpoint**				
30-day all-cause mortality	184	25	0.13 (0.05–0.32)	stage IV 0.58 (0.25–1.32); age (per year) 0.99 (0.96–1.03)
**Exclusions**				
Excluding patients without therapeutic aspiration	173	40	0.15 (0.07–0.31)	—
Excluding monomicrobial *Candida* episodes	178	40	0.15 (0.07–0.30)	—
Excluding contaminant-flagged reports	168	38	0.13 (0.06–0.28)	—
**Separate addition of a fourth covariate**				
log2 C-reactive protein	183	42	0.14 (0.07–0.29)	1.00 (0.84–1.20), p=0.98
log2 procalcitonin	171	37	0.13 (0.06–0.29)	0.96 (0.83–1.12), p=0.61
Neutropenia (<1.5×10^9/L; 6 patients)‡	184	42	0.13 (0.06–0.27)	6.26 (1.37–28.49), p=0.018
Creatinine (per mg/dL)	184	42	0.15 (0.07–0.31)	1.10 (0.71–1.70), p=0.68
Any comorbidity	184	42	0.14 (0.07–0.28)	0.77 (0.38–1.54), p=0.46
Abscess size (per cm)	176	40	0.15 (0.08–0.30)	0.98 (0.91–1.05), p=0.51
≥2 aspiration/drainage sessions	184	42	0.14 (0.07–0.28)	0.88 (0.45–1.69), p=0.70
Hepatic location	184	42	0.14 (0.07–0.27)	0.70 (0.37–1.33), p=0.28
Anatomical site (reference hepatic)§	184	42	0.14 (0.07–0.28)	non-hepatic intra-abdominal 1.71 (0.85–3.44); other 1.04 (0.43–2.51)
Intensive-care admission	178	41	0.14 (0.07–0.28)	0.69 (0.36–1.33), p=0.27
Mechanical ventilation	176	41	0.14 (0.07–0.30)	1.46 (0.52–4.14), p=0.47
Vasopressor use	176	41	0.14 (0.07–0.29)	1.16 (0.55–2.48), p=0.69
Systemic corticosteroid exposure	177	41	0.15 (0.07–0.30)	1.19 (0.55–2.58), p=0.65
Prior biliary or other interventional procedure	184	42	0.12 (0.05–0.26)	0.65 (0.31–1.37), p=0.26
Systemic therapy within 30 days	184	42	0.14 (0.07–0.28)	0.74 (0.17–3.17), p=0.68
Systemic therapy within 90 days	184	42	0.14 (0.07–0.28)	0.93 (0.38–2.28), p=0.88
Any antitumour treatment	184	42	0.14 (0.07–0.29)	1.00 (0.46–2.19), p=0.99
ESBL-producing Enterobacterales	184	42	0.14 (0.07–0.28)	0.96 (0.46–2.00), p=0.91
**Functional form**				
Albumin as 4-df B-spline versus linear	184	42	—	likelihood-ratio p=0.99

CI, confidence interval; HR, hazard ratio; ESBL, extended-spectrum beta-lactamase. ‡Estimate based on six neutropenic patients; the confidence interval is very wide and the estimate should not be interpreted as corroborating evidence. §Age per year in this model.

Table S2. HALP fitted in the primary stratified Cox model (n=184, 42 events)

Model	Variable	HR (95% CI)	p	Apparent C-index	Optimism-corrected C-index	AIC
Albumin model (primary)	Albumin, per 1 g/dL	0.14 (0.07–0.28)	<0.001	0.773	0.762	341.4
	Stage IV	0.70 (0.37–1.33)	0.278			
	Age, per 10 years	1.16 (0.89–1.52)	0.280			
HALP model (albumin excluded)	HALP, per doubling	0.79 (0.60–1.05)	0.101	0.604	0.572	378.1
	Stage IV	0.68 (0.37–1.28)	0.233			
	Age, per 10 years	1.30 (0.99–1.70)	0.060			
Age and stage only	Stage IV	0.70 (0.38–1.30)	0.262	0.584	0.562	378.9
	Age, per 10 years	1.29 (0.98–1.70)	0.064			

Difference in apparent C-index, albumin model minus HALP model: 0.169 (bootstrap 95% CI 0.078–0.247; 1,000 resamples). HALP = haemoglobin (g/L) × albumin (g/L) × lymphocytes (10^9/L) / platelets (10^9/L), log2-transformed. AIC, Akaike information criterion.

Table S3. Wording of the index radiology report (n=183; one report unavailable)

Report wording	n (%)
“Abscess” stated, unqualified	29 (15.8)
“Abscess?” or “compatible with abscess”	36 (19.7)
“Collection” without the word abscess	96 (52.5)
— described as loculated	64
— described as having dense/thick content	25
Cystic or mass-like lesion with query of abscess	8 (4.4)
Other wording (hyperechoic area, soft-tissue increase)	14 (7.7)

Categories are based on manual review of the report text; a report may be counted under more than one descriptive sub-category (loculated, dense content). The word “abscess” was not required for inclusion; the operational case definition is given in Section 2.2.

Table S4. Site-specific characteristics, microbiology and outcomes

	Hepatic	Non-hepatic intra-abdominal	Retroperitoneal	Extra-abdominal	Pelvic	Abdominal wall
Patients, n	73	66	20	11	10	4
Age, median, years	61	61.5	63.5	65	57.5	62
Stage IV, n (%)	45 (62)	27 (41)	10 (50)	6 (55)	7 (70)	4 (100)
Multiple abscesses, n (%)	21 (29)	17 (26)	3 (15)	0 (0)	1 (10)	0 (0)
Abscess size, median, cm	8.0	6.4	7.2	4.0	10.5	5.2
Therapeutic percutaneous aspiration or drainage, n (%)	72 (99)	63 (95)	19 (95)	7 (64)	8 (80)	4 (100)
≥2 sessions, n (%)	30 (41)	13 (20)	4 (20)	1 (9)	3 (30)	1 (25)
Organism recorded, n (%)	52 (71)	48 (73)	11 (55)	5 (45)	6 (60)	2 (50)
Probable pathogen, n	47	41	10	2	6	2
Gram-negative, n (% of recorded)	41 (79)	33 (69)	6 (55)	0 (0)	3 (50)	2 (100)
ESBL-producing / Enterobacterales, n/N	20/39	11/31	5/5	0/0	1/3	1/2
MDR or XDR / gram-negative, n/N	34/41	26/33	5/6	0/0	2/3	2/2
Enterococcus spp., n	4	3	3	0	2	0
Candida albicans, n	2	4	0	0	0	0
Albumin, median, g/dL	2.9	3.0	3.25	3.4	2.9	2.95
Intensive-care admission, n/N	30/68	23/65	2/20	0/11	5/10	1/4
Antibiotic duration, median, days	19.5	19	11	11	10	13.5
Hospital stay, median, days	30	36.5	23	21	26	52
Deaths ≤30 days, n	11	11	1	2	0	0
Deaths ≤90 days, n	15	19	3	3	1	1
30-day mortality, Kaplan–Meier, %	15.2	16.9	5.0	18.2	0.0	0.0
90-day mortality, Kaplan–Meier, %	20.9	29.5	16.2	27.3	11.1	25.0

ESBL, extended-spectrum beta-lactamase; MDR, multidrug-resistant; XDR, extensively drug-resistant. Organism counts include all recorded organisms; probable pathogens exclude the 16 adjudicated contaminants. Univariable Cox model, hepatic versus non-hepatic location: HR 0.85 (95% CI 0.45–1.59; p=0.60). Because several site groups contain very few events, comparisons are descriptive.
